# Virulence and Antimicrobial Resistance Characterization of *Glaesserella parasuis* Isolates Recovered from Spanish Swine Farms

**DOI:** 10.3390/antibiotics13080741

**Published:** 2024-08-06

**Authors:** Alba González-Fernández, Oscar Mencía-Ares, María José García-Iglesias, Máximo Petrocchi-Rilo, Rubén Miguélez-Pérez, César Bernardo Gutiérrez-Martín, Sonia Martínez-Martínez

**Affiliations:** Department of Animal Health, Faculty of Veterinary, Universidad de León, 24007 León, Spain; algonf@unileon.es (A.G.-F.); mjgari@unileon.es (M.J.G.-I.); mpetr@unileon.es (M.P.-R.); rmigp@unileon.es (R.M.-P.); cbgutm@unileon.es (C.B.G.-M.); smarm@unileon.es (S.M.-M.)

**Keywords:** antimicrobial susceptibility profiling, *Glaesserella parasuis*, *Haemophilus parasuis*, pathotyping, pig, porcine respiratory disease complex, TbpB clustering, *vtaA* genes

## Abstract

*Glaesserella* (*Haemophilus*) *parasuis*, the causative agent of Glässer’s disease, is present in most pig farms as an early colonizer of the upper respiratory tract. It exhibits remarkable variability in virulence and antimicrobial resistance (AMR), with virulent strains capable of inducing respiratory or systemic disease. This study aimed to characterize the virulence and the AMR profiles in 65 *G. parasuis* isolates recovered from Spanish swine farms. Virulence was assessed using multiplex leader sequence (LS)-PCR targeting *vtaA* genes, with all isolates identified as clinical (presumed virulent). Pathotyping based on ten pangenome genes revealed the virulent HPS_22970 as the most frequent (83.1%). Diverse pathotype profiles were observed, with 29 unique gene combinations and two isolates carrying only potentially non-virulent pangenome genes. AMR phenotyping showed widespread resistance, with 63.3% classified as multidrug resistant, and high resistance to clindamycin (98.3%) and tylosin (93.3%). A very strong association was found between certain pathotype genes and AMR phenotypes, notably between the virulent HPS_22970 and tetracycline resistance (*p* < 0.001; Φ = 0.58). This study reveals the wide diversity and complexity of *G. parasuis* pathogenicity and AMR phenotype, emphasizing the need for the targeted characterization of clinical isolates to ensure appropriate antimicrobial treatments and the implementation of prophylactic measures against virulent strains.

## 1. Introduction

*Glaesserella parasuis*, formerly known as *Haemophilus parasuis*, a Gram-negative bacterium of the *Pasteurellaceae* family, is present in most commercial pig farms as an early colonizer of the upper respiratory tract in piglets [1]. It is a heterogeneous species that comprises strains with wide differences in virulence [2]. Non-virulent strains constitute a relevant part of the normal nasal microbiota of piglets, while virulent strains are responsible for inducing respiratory or systemic disease [3]. *G. parasuis* virulent strains are the primary pathogens and the cause of Glässer’s disease, a systemic process on weaning piglets characterized by arthritis, meningitis, and polyserositis [2], and a secondary agent in the porcine respiratory disease complex (PRDC) [4].

Glässer’s disease poses a significant economic challenge to swine production worldwide, with major impact in nursery and early fattening stages. Acute outbreaks of the disease can result in high mortality rates, ranging from 5 to 10%, especially in young animals [3]. This, together with its role in complicating primary infections in the PRDC, further impacts production parameters. This leads to substantial economic losses due to reduced growth performance, the need for carcass disposal, and the costs associated with treatments [5]. Unfortunately, the precise economic burden of *G. parasuis* has not been fully quantified [3].

The epidemiology of *G. parasuis* is primarily assessed through serotyping, which is based on capsular polysaccharides. To date, 15 serotypes have been identified [6]. The wide distribution of these serotypes worldwide, found both in virulent and non-virulent strains, underscores *G. parasuis* heterogeneity. For instance, a recent study in North America reported 11 different serotypes, with serotypes 7, 13, 2, 4, and 5 being the most prevalent [7]. These serotypes are also commonly detected in clinical samples from North and South America, Asia, and Europe [8,9,10]. However, in many regions serotypes 4 and 5 are the most frequently identified, as reported in studies from Brazil, Germany, China, or Australia [11,12,13,14]. In Spain, the most recent study reported nine different *G. parasuis* serotypes in clinical cases, with serotypes 5, 10, 2, and 4 being the most frequent [15]. These epidemiological findings highlight the genomic complexity of *G. parasuis* both among and within regions, necessitating the specific assessment of the virulence of each isolate to ensure therapeutic and management strategies.

The pathogenic variability observed among *G. parasuis* strains urges for the precise molecular characterization for effective disease management. Although serotyping was initially deemed a reliable indicator of virulence [16], recent studies have challenged its usefulness [10,17], prompting the exploration for alternative biomarkers. The virulence-associated trimeric autotransporter genes (*vtaA*) [18] and pathotyping based on the characterization of ten pangenome genes [19] have been proposed as reliable indicators of *G. parasuis* pathogenicity. Additionally, the sequencing of the transferrin-binding protein B gene (*tbpB*) has been demonstrated as promising in stratifying *G. parasuis* into three clusters based on its virulence potential [20].

Addressing Glässer’s disease in swine production does not only entail determining the virulence of the *G. parasuis* isolates recovered from the farm, but also evaluating their antimicrobial resistance (AMR). Beta-lactams, phenicols, macrolides, potentiated sulfamides, and tetracyclines have been described to be effective to treat this disease [21]. However, due to the overuse and misuse of antimicrobials on pig farms, a remarkable increase in AMR among pathogenic *G. parasuis* has been observed in recent years [3]. Therefore, this study aims to characterize the virulence and antimicrobial susceptibility profiling of a selection of *G. parasuis* isolates recovered from Spanish pig farms. By establishing an association among pathogenicity biomarkers and AMR patterns, we seek to enhance the understanding and management of Glässer’s disease, contributing to more targeted and effective therapeutic strategies.

## 2. Results

### 2.1. Characterization of Virulence and Pathotyping in Glaesserella parasuis Isolates

According to the virulence status based on the amplification of *vtaA* genes, all 65 *G. parasuis* isolates included in the study were defined as clinical (presumed virulent), with no isolate characterized as non-virulent.

**Figure 1:**
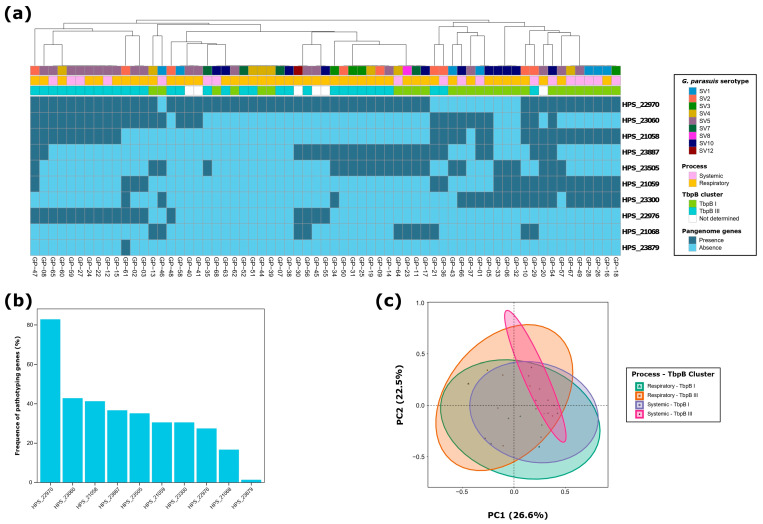
Pathotype characterization of 65 *G. parasuis* isolates from Spanish swine farms: (**a**) pathotype clustering based on the presence of ten pangenome genes, using the unweighted pair group method with arithmetic mean (UPGMA) as the hierarchical clustering method; (**b**) frequency (%) of each pathotype gene; and (**c**) principal component analysis (PCA) of the ten evaluated pangenome genes, showing grouping based on pathologic process and TbpB cluster of each *G. parasuis* isolate.

In the pathotype characterization, the virulent HPS_22970 gene was the most frequently identified, present in 54 of the 65 isolates (83.1%). The frequence of seven other pangenome genes ranged between 43% and 27%, highlighting a diverse virulence profile across the isolates (Figure 1a). The BAPS4 HPS_23879 gene was the least common, detected in only one isolate (Figure 1b).

Further analysis revealed the broad heterogeneity in the combination of pathotype genes within the isolates. We identified 29 different gene combinations (Supplementary Table S1), including 18 unique combinations that were exclusive to individual isolates. The most common pattern involved the presence of only the virulent HPS_22970 gene in nine isolates (13.9%). Similarly, the combination of virulent HPS_21058 and HPS_22970, carriage HPS_23060, and BAPS4 and BAPS5 HPS_22976 genes was frequent, accounting for 12.3% (*n* = 8) of *G. parasuis* isolates. Considering both virulent and BAPS genes as potentially virulent, we detected that 35.4% of *G. parasuis* isolates (*n* = 23) carried three of these five presumably virulent genes, with only one isolate harboring four of these genes. Notably, we detected two presumably carriage *G. parasuis* isolates, not harboring any potentially virulent pangenome gene, which may contradict the results of the *vtaA* genes that consider all *G. parasuis* isolates to be virulent.

The analysis of the association between pathotype genes and the TbpB clustering to which each *G. parasuis* isolate was assigned demonstrated that virulent HPS_22970 and BAPS4 and BAPS5 HPS_22976 were significantly more frequent in bacteria belonging to the TbpB cluster III (*p* < 0.05). Conversely, the carriage HPS_23300 gene showed a significant association with isolates from the TbpB cluster I (*p* < 0.001). When examining the link between pathotyping and the pathologic process, we observed a significantly higher frequence of isolates recovered from piglets exhibiting systemic symptoms carrying the virulent HPS_21058 and carriage HPS_23300 genes (*p* < 0.05). No significant association was found for the serotype of the isolates and their geographic origin.

The ordination of *G. parasuis* isolates based on their pathotype genes showed that the first two dimensions of the PCA represented 49.1% of the variability (Figure 1c). Dimension 1 represented 26.6% of the variability, and it was mainly determined by HPS_21058 (29.8%) and HPS_23060 (23.1%) genes. Dimension 2 included 22.5% of the variability, and it was predominantly determined by the HPS_23300 (30.5%) and HPS_22976 (22.3%) genes. Remarkably, the ordination based on the TbpB clustering, and the pathologic process explained 9.6% and 4.2% of the variability observed among *G. parasuis* isolates, respectively (adonis2, *p* < 0.05). No effect of the serotype or the geographic origin of the isolates was appreciated.

### 2.2. Antimicrobial Susceptibility Profiling of Glaesserella parasuis Isolates

**Figure 2:**
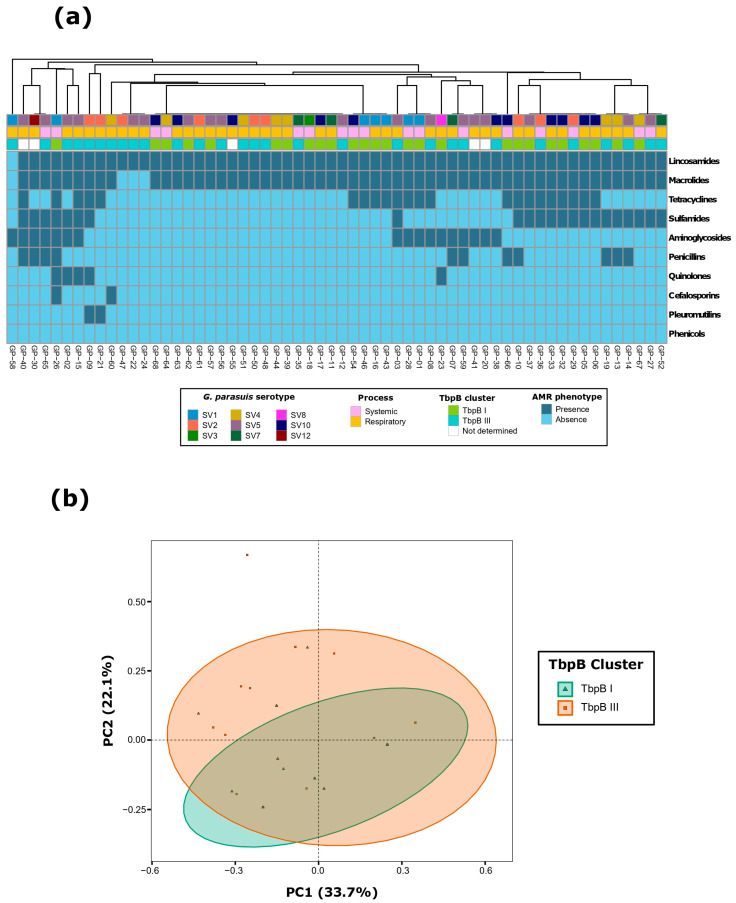
Antimicrobial resistance (AMR) characterization at the class level of 60 *G. parasuis* isolates from Spanish swine farms: (**a**) AMR phenotype clustering based on antimicrobial classes, using the unweighted pair group method with arithmetic mean (UPGMA) as the hierarchical clustering method, and (**b**) principal component analysis (PCA) of the AMR patterns, showing grouping based on the TbpB cluster of each *G. parasuis* isolate.

A total of 60 *G. parasuis* isolates were evaluated for their AMR phenotype. MIC values for all antimicrobials are shown in Table 1. All isolates were resistant to at least one antimicrobial class, with thirty-eight isolates (63.3%) defined as MDR, nine with resistance to more than five classes (15.0%), and only one isolate resistant to eight classes (Figure 2a, Table 2). There were 22 AMR patterns (Supplementary Table S2), with the combination of macrolides and lincosamides being the most common (30%, *n* = 18), followed by the combination of tetracyclines, sulfamides, macrolides, and lincosamides (11.6%, *n* = 7). Non-susceptible phenotype was mainly detected for clindamycin (98.3%, *n* = 59) and tylosin (93.3%, *n* = 56), followed by sulfadimethoxine (36.7%, *n* = 22), chlortetracycline (36.7%, *n* = 22), and penicillin (16.6%, *n* = 10). Particularly remarkable are the differences within the macrolides class, contrasting the high AMR detected for tylosin with the complete susceptibility for tulathromycin and tilmicosin. No isolates were resistant to either florfenicol and danofloxacin. The only demonstrated association between the TbpB clustering to which each *G. parasuis* isolate was assigned and their AMR phenotype was the significantly higher tetracycline resistance in isolates from the TbpB cluster I (*p* < 0.05). No significant association was found for the pathologic process, the serotype, or the geographic origin of the isolates.

The analysis of *G. parasuis* isolates based on their AMR phenotype at the class level revealed that the first two dimensions of the PCA captured 55.8% of the total variability (Figure 2b). Dimension 1 accounted for 33.7% of the variability, primarily influenced by sulfonamides (46.5%) and tetracyclines (32.7%). In contrast, Dimension 2 explained 22.1% of the variability, with significant contributions from aminoglycosides (57.6%), tetracyclines (22.4%), and penicillins (15.9%). Additionally, when considering the TbpB clustering, it accounted for 4.0% of the observed variability among *G. parasuis* isolates (adonis2, *p* < 0.05). There was no discernible impact from the pathologic process, serotype, or geographic origin.

### 2.3. Association between Pathotypes and Antimicrobial Susceptibility Profiling of Glaesserella parasuis Isolates

**Figure 3:**
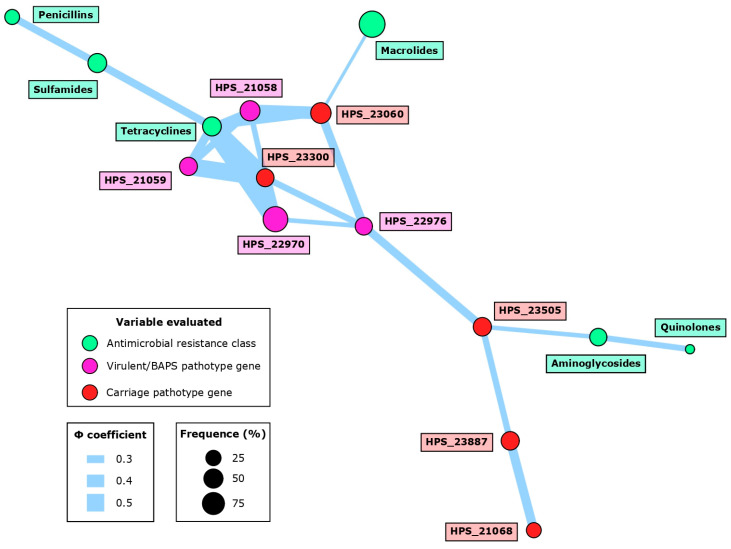
Network associations between pathotype genes and antimicrobial resistance (AMR) at the class level in 60 *G. parasuis* isolates from Spanish swine farms. Node size is determined by the percentage occurrence of the pathotype gene or AMR class. Edge size is proportional to the magnitude of the association based on the Φ coefficient. The network was constructed using significant associations (*p* < 0.05).

Several associations were observed between pathotype genes and the AMR phenotypes (Figure 3, Table 3). The most frequent interaction was the cooccurrence of tetracycline resistance with five different pangenome genes, highlighting the very strong association (Φ > 0.25) with virulent HPS_22970 (*p* < 0.001; Φ = 0.58) and carriage HPS_23300 (*p* < 0.001; Φ = 0.52). Carriage HPS_23060 exhibited a significant association with tetracycline (*p* < 0.05; Φ = 0.29) and macrolide resistance (*p* < 0.05; Φ = 0.22). Interestingly, we also observed a very strong cooccurrence between carriage HPS_23505 and aminoglycoside resistance (*p* < 0.05; Φ = 0.25).

Within pathotype genes, 11 significant cooccurrences were appreciated, with the notable association between carriage HPS_23300 and virulent HPS_21059 (*p* < 0.0001; Φ = 0.53) or between carriage HPS_23060 and virulent HPS_21058 (*p* < 0.001; Φ = 0.43). We could also determine three associations between AMR phenotypes, remarking the interaction between sulfonamides and tetracyclines (*p* < 0.05; Φ = 0.32). All these findings demonstrate the complexity of the interactions between AMR and pathotype genes.

## 3. Discussion

*G. parasuis* is a significant pathogen in swine production, causing substantial economic losses by affecting pig health and productivity [3]. Its extensive heterogeneity, attributed to a wide pangenome [7,22], with the simultaneous presence of virulent and non-virulent *G. parasuis* strains in the respiratory tract, necessitates the implementation of diagnostic tools to accurately identify virulent strains and determine its antimicrobial susceptibility to ensure therapeutic success. This study highlights the usefulness of currently proposed virulence indicators for identifying pathogenic strains and underscores the wide diversity of virulent *G. parasuis* isolates in Spanish swine farms. These findings reveal the necessity for tailored diagnostics for effective *G. parasuis* control on each swine farm.

Several factors have been described to contribute to disease development, including the presence of other pathogens on the farm and the virulence of *G. parasuis* strains [23]. Nearly 150 genes have been recently identified as potentially involved in *G. parasuis* virulence, although their strict association with pathogenicity remains unproven [22]. Among these, the *vtaA* genes, proposed as virulence indicators [18], are essential for *G. parasuis* survival in the lungs by resisting phagocytosis from alveolar macrophages [24]. In this study, the multiplex leader sequence (LS)-PCR used to amplify *vtaA* genes confirmed the virulence of all tested *G. parasuis* isolates, consistent with previous studies evaluating presumably virulent isolates from diseased animals [11,12,25]. Since all *G. parasuis* isolates in this study were recovered from clinical samples, this finding underscores the usefulness of the LS-PCR as a rapid and reliable predictor of *G. parasuis* virulence.

The *G. parasuis* pathotype, based on ten putative virulence-related sequences [19], revealed 29 different gene combinations, as previously observed [12], highlighting the significant role of the accessory genome in *G. parasuis* pathogenicity [22]. This approach enables the classification of *G. parasuis* isolates into virulent, potentially virulent, and non-virulent categories based on pangenome gene combination. However, the need for an online tool or a software application to predict this classification presents a challenge, as these resources are not currently accessible to the general public, complicating precise virulence predictions using the pathotyping PCR method outlined by Howell et al. [19]. Despite this limitation, an individual evaluation of pangenome genes showed that only two *G. parasuis* isolates lacked potentially virulent pangenome genes, which might contrast with the LS-PCR *vtaA* results. Schuwerk et al. [12] demonstrated that strains without potentially virulent pangenome genes might still be classified as virulent using the pathotyping model, complicating its interpretation. Therefore, further refinement and validation of pathotyping PCR are necessary to ensure its reliability and ease of interpretation in different contexts.

This study suggests a potential association between the pathological process and the TbpB cluster with the pathotype gene combination, emphasizing the multifaceted nature of *G. parasuis* virulence mechanisms. Although previous studies by Schuwerk et al. [12] and Howell et al. [19] could not distinguish systemic from respiratory *G. parasuis* isolates based on gene combinations, individual markers and genes were identified in systemic isolates. These findings imply that certain *G. parasuis* strains may be adapted to specific locations, likely due to its accessory genome. In addition, we observed no association between *G. parasuis* serotype and pathotype, aligning with previous studies [12,26]. This lack of relationship underscores the complexity of determining virulence through serotyping alone, emphasizing the need for comprehensive virulence characterization to identify virulent *G. parasuis* strains accurately.

Despite the current success in reducing antimicrobial use in swine production in Europe and, particularly, in Spain [27], antimicrobials remain essential for the treatment of Glässer’s disease due to the lack of a universal vaccine for its prevention and control [28]. Consequently, evaluating the phenotypic AMR profile of virulent *G. parasuis* isolates remains crucial. In this study, we observed a remarkably high rate of MDR (63.3%) *G. parasuis* on Spanish swine farms. However, most commonly used antimicrobials were effective. Notably, AMR was particularly high for clindamycin (98.3%) and tylosin (93.3%), while resistance rates for other antimicrobials remained below 40%. This finding is significant as the European Medicines Agency (EMA) categorizes these antibiotics for veterinary use into category C (caution, first option) and D (prudence, second option) [29], ensuring a broad therapeutic availability against this pathogen in swine production. In contrast, regions like China [30] or Brazil [11] face higher AMR rates for antimicrobials categorized as category B (restrict, last resort antimicrobials in veterinary medicine), such as quinolones, which exhibited an AMR rate of below 9% in this study.

The observed high tylosin resistance aligns with previous reports [11,31], as this antimicrobial is frequently used to control Glässer’s disease outbreaks [32]. *G. parasuis* can also develop cross-resistance between macrolides and lincosamides, for instance, through plasmids carrying the *lnu(C)* antimicrobial resistance gene (ARG) [33] or the *erm(A)* ARG [7]. This may explain the high clindamycin resistance observed, which has also been reported in previous studies [11,31]. Indeed, 93% of *G. parasuis* isolates in this study were co-resistant to both antimicrobials. Interestingly, despite the high resistance to tylosin, no resistance was observed for other macrolides, i.e., tulathromycin and tilmicosin. This might be due to specific AMR mechanisms or ARGs conferring resistance to the first generation of macrolides like tylosin. Given the limited number of detailed molecular studies regarding AMR mechanisms in *G. parasuis* [7,22,31], further research is critical to establish associations between phenotypic and genotypic results.

The increased selective pressure of AMR due to the overuse and misuse of antimicrobials, coupled with environmental factors, has contributed to the selection and dissemination of certain bacterial strains [34]. The co-location of genes in specific genomic regions, especially within mobile genetic elements, can contribute to the co-selection of virulence genes and ARGs [35]. This study highlights a strong statistical association between certain pathotype genes and AMR phenotypes in *G. parasuis*. Notably, tetracycline resistance showed a consistent association with specific virulent pathotype genes, such as HPS_22970 (Φ = 0.58) and HPS_21058 (Φ = 0.28). To the best of our knowledge, this is the first study to suggest an association between AMR and virulence in *G. parasuis*. This may be attributed to the high genetic variation within *G. parasuis* and the presence of most pathogenic genes [22] and ARGs [36] in the accessory genome. Indeed, associations between certain *G. parasuis* lineages and ARGs have been reported [7]. Therefore, further genomic investigations are essential not only to determine the underlying mechanisms of AMR but also the role of the accessory genome and the structure of its mobilome in the co-selection and dissemination of virulence and AMR in *G. parasuis*.

In conclusion, this study provides significant insight into the wide diversity and complexity of virulence and AMR of *G. parasuis* isolates from Spanish swine farms, highlighting the importance of accurate diagnostic tools to ensure therapeutic success. The *vtaA* LS-PCR is shown to be an effective and easy predictor of *G. parasuis* virulence, and its combination with a more accessible pathotyping PCR could enhance virulence characterization. The observed diversity in pathotyping and AMR profiles underscores the heterogeneity of *G. parasuis* and the essential role of the accessory genome. Despite the high rate of MDR, most antimicrobials remain effective against clinical *G. parasuis* on Spanish swine farms. Accurate characterization is essential to prevent AMR development and dissemination. Notably, associations observed between specific pathotype genes and AMR phenotypes suggest a complex interplay between virulence and resistance mechanisms. These findings underscore the need for ongoing genomic research to better understand the co-selection and dissemination of virulence and AMR, ultimately contributing to more effective control strategies for Glässer’s disease in swine production.

## 4. Materials and Methods

### 4.1. Selection of Glaesserella parasuis Isolates Recovered from Spanish Pig Farms

A selection of 65 isolates of *G. parasuis*, previously characterized for their serotype and phylogenetic analysis [15], was used in this study (Supplementary Table S3). Briefly, these isolates were recovered from weaning piglets presenting respiratory disease (*n* = 47) or systemic symptoms of Glässer’s disease (*n* = 18) on Spanish pig farms between 2018 and 2021.

### 4.2. Glaesserella parasuis Growth Conditions and DNA Extraction

For all analyses that required *G. parasuis* growth, isolates were cultured on chocolate agar plates (Thermo Scientific, Oxoid, UK), under microaerophilic conditions at a temperature of 37 °C for at least 48 h.

For further virulence characterization, the DNA extraction of *G. parasuis* isolates was performed. A single colony was inoculated in 100 µL sterile distilled water and boiled for 10 min at 100 °C. Subsequently, it was centrifuged at 12,000 rpm for 10 min, and the supernatant with the extracted DNA was transferred to a new sterile microtube for further analyses. Purified supernatant was stored at −20 °C until further use.

### 4.3. Virulence Characterization of Glaesserella parasuis Isolates

For determining the virulence status, an LS-PCR was performed to amplify the *vtaA* genes, adapting the protocol from Galofré-Milá et al. [18]. Specifically, the LS-PCR utilized the forward LS-primer AV1-F (5′ AAATATTTAGAGTTATTTGGAGTC 3′) for common amplification. To differentiate the virulence status, two reverse LS-primers were used, i.e., V1-R (5′ AATATACCTAGTAATACTAGACTTAAAAG 3′), targeting *vtaA* in clinical (presumed virulent) strains, and NV1-R (5′ CAGAATAAGCAAAATCAGC 3′), for non-clinical (non-virulent) strains. The differentiation between strains was based on amplicon size, with clinical isolates (V1-R) yielding 200 base pairs (bp) and non-clinical isolates (NV1-R) yielding 300 bp.

The amplification PCR protocol included a denaturalization step at 94 °C for 5 min, followed by 30 amplification cycles that included a 45 s denaturalization at 94 °C, a 45 s annealing at 52 °C, and a 1 min extension at 72 °C with a final extension at 72 °C for 7 min.

### 4.4. Pathotype Characterization of Glaesserella parasuis Isolates

Pathotyping of the *G. parasuis* isolates followed the methodology of Howell et al. [19] that utilized a selection of ten pangenome genes, which were categorized as virulent, carriage, or Bayesian analysis of population structure (BAPS). This characterization was divided into three multiplex PCR panels: Panel I included genes HPS_21058 (virulent), HPS_21059 (virulent), HPS_22970 (virulent), HPS_23300 (carriage), and HPS_23887 (carriage); Panel II included genes HPS_21068 (carriage), HPS_23060 (carriage), and HPS_23505 (carriage); and Panel III comprised genes HPS_23879 (BAPS4) and HPS_22976 (BAPS4 and BAPS5). Primers used in the pathotyping PCR are available in Supplementary Table S4. In all PCR assays, the virulent reference strain *G. parasuis* Nagasaki and nuclease-free water were included as positive and negative controls, respectively.

The three multiplex amplification protocols included a denaturalization step at 94 °C for 1 min, followed by 30 amplification cycles that included a 30 s denaturalization at 94 °C, a 30 s annealing at 54 °C, and a 1 min extension at 68 °C with a final extension at 68 °C for 5 min.

### 4.5. Antimicrobial Susceptibility Profiling of Glaesserella parasuis Isolates

Antimicrobial susceptibility testing was conducted on 60 out of the 65 recovered *G. parasuis* isolates. The determination of the minimum inhibitory concentration (MIC) followed the broth microdilution technique outlined by Prüller et al. [37], in accordance with the recommendations provided by the Clinical and Laboratory Standards Institute (CLSI). Clinical breakpoints (susceptible and non-susceptible) were set primarily based on the information provided in the CLSI VET01S document [38] and further completed with the CLSI performance standard M100 [39] and breakpoints defined in previous studies [11]. Intermediate and resistant values were included into non-susceptible phenotypes. Non-susceptible and resistant will be used indistinctly throughout the study. Multidrug resistance (MDR) was defined as acquired non-susceptibility to at least one agent in three or more antimicrobial classes [40]. A microorganism susceptible to all antimicrobials tested was defined as pansusceptible (PNS).

AMR was evaluated with the commercial Bovine/Porcine BOPO6F Sensititre plates (TREK Diagnostic Systems, East Grinstead, UK), with the Nagasaki strain as the control strain. The evaluated antimicrobials and their breakpoints are shown in Table 1.

Prior to testing, *G. parasuis* colonies from a fresh culture were suspended in 5 mL of 0.9% saline and adjusted to a turbidity comparable to that of a 0.5 McFarland standard. This bacterial suspension was then diluted 1:200 in cation-adjusted Mueller–Hinton broth (Thermo Fisher Scientific, Waltham, MA, USA) supplemented with 0.0025% NADH (Sigma-Aldrich, St. Louis, MO, USA) and 1% sterile filtered and heat-inactivated chicken serum (Gibco, Norristown, PA, USA), to reach a density of 5 × 10^5^ CFU/mL. Then, 50 µL per well of this suspension was dispensed with the Sensititre AIM Automated Inoculation Delivery System (TREK Diagnostic Systems, East Grinstead, UK). Plates were sealed and incubated in a microaerophilic atmosphere at 37 °C for 48 h.

### 4.6. Statistical Analysis and Figure Visualization

A database was created in an Excel sheet (Microsoft Office 365) to include TbpB clustering (I or III), pathologic process (systemic or respiratory), serotyping, and geographic origin (Spanish province) for each *G. parasuis* isolate, combined with the pathotype genes (presence or absence) and each antimicrobial tested (susceptible or resistant). AMR analyses were performed at the antimicrobial and antimicrobial class levels. All analyses were conducted using R version 4.3.2 (2023-10-31 ucrt) [41], with significance established at *p* < 0.05. Plots were produced using *ggplot2* 3.5.1, *igraph* 2.0.3, and *ggraph* 2.2.1 packages [42,43,44], and further modified using the software Inkscape version 1.3.2 (https://inkscape.org/, accessed on 23 June 2024).

A clustering of *G. parasuis* isolates was performed according to their pathotype and their AMR phenotype using the unweighted pair group method with arithmetic mean (UPGMA) as the hierarchical clustering method. The *pheatmap* 1.0.12 package [45] was used for the representation of the clustered heatmaps of isolates. Both pathotype genes and AMR phenotype comparisons among isolates from different TbpB clusters, pathologic processes, serotypes, and geographic origins were carried out with the Fisher’s exact test. *p*-values were adjusted following the Benjamini and Hochberg method [46].

Ordination of *G. parasuis* isolates based on their pathotype and AMR phenotype was estimated using Jaccard distance matrix and analyzed by principal component analysis (PCA), and the two main dimensions for the principal components were characterized. The effect of the TbpB clustering, pathologic process, serotyping, and geographic origin on isolate dissimilarities was determined by permutational multivariate analysis of variance (PERMANOVA) using distance matrices with “*adonis2*” function (pairwise adonis).

To evaluate the association between pathotype genes and the AMR phenotype, a comprehensive methodology was employed. The primary approach involved the execution of the Fisher’s exact test to identify significant associations between genes and AMR profiles, complemented by the calculation of the Phi coefficient (Φ) to measure the strength of these associations. Subsequently, the percentage of occurrence for each gene and antimicrobial was computed. Significant associations were visualized in a network graph, where node sizes were determined by their percentage occurrence and edge sizes reflected the magnitude of Φ. Φ was categorized as very strong (Φ > 0.25), strong (Φ > 0.15), moderate (Φ > 0.10), or weak (Φ > 0.05).

## Figures and Tables

**Table 1:** Minimum inhibitory concentrations (MICs) of 18 antimicrobials against 60 *Glaesserella parasuis* isolates from Spanish swine farms. The thick line represents the clinical breakpoint used for each antimicrobial to classify isolates into susceptible and non-susceptible. Areas in gray represent values outside the concentrations included in the broth microdilution method.

Antimicrobial	Number of Isolates with MIC (µg/mL)													MIC		Susceptible		Non-Susceptible	
	0.12	0.25	0.5	1	2	4	8	16	32	64	128	256	512	MIC_50_	MIC_90_	*n*	%	*n*	%
Penicillin	31	19	8	2	0	0	0							≤0.12	0.5	50	83.3	10	16.6
Ampicillin		45	10	3	1	0	0	0	1					≤0.25	0.5	55	91.7	5	8.3
Ceftiofur		57	0	0	1	0	1	1						≤0.25	≤0.25	58	96.7	2	3.3
Gentamicin				17	30	10	1	1	1					2	4	47	78.3	13	21.6
Spectinomycin							31	27	1	1				≤8	16	59	98.3	1	1.7
Neomycin						23	29	5	2	1				8	16	52	86.7	8	13.3
Tulathromycin				28	26	5	1	0	0	0				2	4	60	100	0	0
Tilmicosin						57	3	0	0	0				≤4	≤ 4	60	100	0	0
Tylosin			3	1	2	9	16	17	11	1				8	32	4	6.7	56	93.3
Chlortetracycline			38	19	1	1	1							≤0.5	1	38	63.3	22	36.7
Oxytetracycline			54	4	0	0	1	1						≤0.5	1	54	90.0	6	10
Danofloxacin	59	1	0	0										≤0.12	≤0.12	60	100	0	0
Enrofloxacin	55	0	1	3	1									≤0.12	≤0.12	55	91.7	5	8.3
Clindamicin		0	1	7	16	30	6	0						4	8	1	1.7	59	98.3
^a^ SXT					59	1								≤2/38	≤2/38	59	98.3	1	1.7
Sulfadimethoxine												38	22	≤256	>256	38	63.3	22	36.7
Tiamulin			2	4	3	16	26	7	2					8	16	58	96.7	2	3.3
Florfenicol		44	14	1	1	0	0							≤0.25	0.5	60	100	0	0

^a^ SXT: Sulfamethoxazole–Trimethoprim.

**Table 2:** Antimicrobial resistance (AMR) combinations at class level in 60 *Glaesserella parasuis* isolates from Spanish swine farms.

AMR Combinations	Number of Isolates (*n*)	Frequence (%)
Pansusceptible	0	0
1 antimicrobial class	4	6.7
2 antimicrobial classes	18	30
3 antimicrobial classes	11	18.3
4 antimicrobial classes	18	30
5 antimicrobial classes	5	8.3
6 antimicrobial classes	3	5
7 antimicrobial classes	0	0
8 antimicrobial classes	1	1.7

**Table 3:** Significant associations (*p* < 0.05) between pathotype genes and phenotypic antimicrobial resistance (AMR) at class level in 60 *Glaesserella parasuis* isolates from Spanish swine farms.

Pairwise Association	Φ Coefficient	Φ Categorization	*p*-Value
Tetracyclines—HPS_22970	0.58	Very strong	<0.0001
Tetracyclines—HPS_23300	0.52	Very strong	<0.0001
Tetracyclines—HPS_21059	0.34	Very strong	0.008
Tetracyclines—HPS_23060	0.29	Very strong	0.016
Tetracyclines—HPS_21058	0.28	Very strong	0.029
Aminoglycosides—HPS_23505	0.25	Strong	0.034
Macrolides—HPS_23060	0.22	Strong	0.042
HPS_21058—HPS_23060	0.43	Very strong	0.0006
HPS_22970—HPS_23300	0.39	Very strong	0.002
HPS_21068—HPS_23887	0.35	Very strong	0.004
HPS_23060—HPS_22976	0.34	Very strong	0.005
HPS_23505—HPS_22976	0.33	Very strong	0.005
HPS_21058—HPS_21059	0.31	Very strong	0.012
HPS_23300—HPS_22976	0.29	Very strong	0.012
HPS_23505—HPS_23887	0.29	Very strong	0.019
HPS_21058—HPS_23300	0.27	Very strong	0.024
HPS_22970—HPS_22976	0.25	Strong	0.025
Tetracyclines—Sulfamides	0.32	Very strong	0.011
Penicillins—Sulfamides	0.31	Very strong	0.012
Aminoglycosides—Quinolones	0.29	Very strong	0.020

## Data Availability

The original contributions presented in the study are included in the article/Supplementary Materials, and further inquiries can be directed to the corresponding author.

## Supplementary Materials

The following supporting information can be downloaded at: https://www.mdpi.com/article/10.3390/antibiotics13080741/s1, Table S1: Combination of pathotype genes in 65 *G. parasuis* isolates from Spanish swine farms.; Table S2: Combination of phenotypic antimicrobial resistance at the class level in 60 *G. parasuis* isolates from Spanish swine farms; Table S3: Detailed information of the 65 *G. parasuis* isolates included in the study; Table S4: Primers used for pathotype characterization of *G. parasuis*.
