# Supplementary material for: Virulence and Antimicrobial Resistance Characterization of Glaesserella parasuis Isolates Recovered from Spanish Swine Farms
Source: Antibiotics (Basel). 2024 Aug 6;13(8):741. doi: 10.3390/antibiotics13080741 (PMC11350796; doi:10.3390/antibiotics13080741)
Supplement: Supplementary file 1 [file antibiotics-13-00741-s001.zip › Table_S4.pdf]

Supplementary Table S4. Primers used for pathotype characterization of *G. parasuis* [19].

Pathotype gene	Primer sequence (5′- 3′)	Amplicon size (bp)
**HPS_21058**	**F-**CCGAAAGCATAGATCCAAATGC<br>**R-**CCACCTTGTTTACTTGCTTCTGC	590
**HPS_21059**	**F-**CGTAGCATACGCACACCTAAAG<br>**R-**GAAAGGGCAATAGATACATTTCGG	720
**HPS_21068**	**F-**TGATAATGCACAGATAGTGGGTAGCTCT<br>**R-**TATGACTACTCGCAATGAATTGCTCTG	520
**HPS_22970**	**F-**CAAGGAAGTGTTTATTTGGGGAAGAAGG<br>**R-**GCTCGATCCAACCCTGAATTTATC	240
**HPS_23060**	**F-**CCTGTGATTGAATGGGTTCCTCG<br>**R-**GTTGTTTAGATCATCATCCATTAC	300
**HPS_23300**	**F-**GGATATACACTCAGTCTTAGCCCTA<br>**R-**GCAGGTTCTCTTGATTTAGCTTTTC	120
**HPS_23505**	**F-**GTAACAGCTAACCGAGGAAATTGT<br>**R-**TAAGAGTATATGTCCAATCTCCCCG	170
**HPS_23879**	**F-**GGATAACCCTGATGAACTTGATGAAGAGC<br>**R-**CGAATGTTCGGATTTGTTCAAGTTGG	210
**HPS_23887**	**F-**GATTTAGCTGAAGCTCAAGACA<br>**R-**GCAAAGCGACATAACCTGCTGATCTTCTC	470
**HPS_22976**	**F-**ATTGAGCAGATTGAGCAACGGAAAA<br>**R-**TTACCAAAGCCCTAATGTTGGGTTT	375
